# Increased tryptase in children with allergic reactions to hazelnut

**DOI:** 10.1186/2045-7022-5-S3-P108

**Published:** 2015-03-30

**Authors:** Josef Brändström, Niclas Uppsten Rydell, Anders Sjölander, Caroline Nilsson

**Affiliations:** 1 Institute of Clinical Science and Education, Sodersjukhuset and Sachs Children’s Hospital, Sodersjukhuset, Stockholm, Sweden; 2 Thermo Fisher Scientific, Uppsala, Sweden

## Background

Mast cells are activated during allergic reactions, releasing inflammatory mediators that include tryptase. This process leads to the symptoms of an allergic reaction. A transient increase in the total level of serum tryptase indicates mast cell activation, e.g. an anaphylactic reaction. This may help to confirm and assess the extent of a systemic reaction. In the current study, the total level of serum tryptase was measured at different time points; before, during and after double-blind placebo-controlled food challenge (DBPCFC) with hazelnut in 40 suspected hazelnut allergic children aged 6-18 years. Total serum tryptase was measured with ImmunoCAP Tryptase, Thermo Fisher Scientific, Uppsala, Sweden.

## Results

Of the 40 children, eight were positive during the DBPCFC. Of these, five showed an increase in serum tryptase concentrations during or after challenge that was >20% compared to serum tryptase base line levels. Two patients had a >20% increase of tryptase when eating placebo and without reporting any symptoms. In addition, the serum baseline tryptase levels in the children experiencing a clinical reaction were statistically higher compared with the non-responding children (p<0.05).

## Conclusion

Our results show that tryptase measurement can be a valuable objective tool for confirmation of mast cell activation during DBPCFC in children. The results further indicate that measurement of tryptase may have a predictive value as indicator for a systemic response during DBPCFC.

